# A Thematic Analysis of the Patient Centered Outcomes Research Institute’s (PCORI) Stakeholder Identified ADRD Research Priorities

**DOI:** 10.1002/alz.095028

**Published:** 2025-01-09

**Authors:** Joanna Philips, John Audley, Yewande Akinbami, Tabassum Majid, Neeraj Arora, Sarah Penuela‐Wermers

**Affiliations:** ^1^ PCORI, Washington, DC USA

## Abstract

**Background:**

National and international organizations, including the United Nations, Center for Disease Control, Agency for Healthcare Research and Quality, and the US Surgeon General’s Office have highlighted the need for a comprehensive approach to caring for the growing number of older adults in the U.S. Furthermore, advancements in both non‐pharmacological and pharmacological treatments for Alzheimer’s Disease and Related Dementias (ADRD), coupled with the CMS initiative on improving dementia care management (GUIDE), have intensified national attention on the needs of older adults living with ADRD. Acknowledging this convergence of factors and with the input of stakeholders, PCORI identified “Promoting the Health of Older Adults” as one of 12 topic themes for future research. As the leading funder of Clinical Comparative Effective Research (CER), PCORI has invested $88 million to support 15 CER studies focused on ADRD and 10 awards on engagement related initiatives to advance ADRD research interests.

**Method:**

To further identify funding gaps and understand CER priority areas related to ADRD, we conducted in‐depth analyses of PCORI’s ADRD portfolio and of discussions on ADRD with diverse stakeholder partners (i.e. patients, clinicians, researchers, nursing organizations, payers, advocacy groups). An analysis of 24 ADRD‐related stakeholder convenings using open‐ended unstructured interview questions was done and common themes of priority CER areas were identified.

**Result:**

The thematic analysis revealed three ADRD CER priority areas related to: (1) Sensory impairment in ADRD patients, (2) Coordination of healthcare services and (3) Support for caregivers of ADRD patients. Moreover, stakeholders identified the need for interventions that address health inequities in underserved populations affected by ADRD and innovative approaches to navigating a fragmented health care system highlighted by the COVID‐19 pandemic.

**Conclusion:**

Evaluation of PCORI’s ADRD portfolio and engagement discussions provide important insights on current critical evidence gaps and real‐world decisional dilemmas. The engagement discussions illuminate priority areas for research that will holistically address those impacted by ADRD at the patient, caregiver, health system, and community level. These insights reveal a need for research that will focus on underserved older adults and their caregivers, as well as optimizing the coordination and integration of care for this population.

